# Clinical course and need for hospital admission after lithium discontinuation in patients with bipolar disorder type I or II: mirror-image study based on the LiSIE retrospective cohort

**DOI:** 10.1192/bjo.2019.83

**Published:** 2019-11-22

**Authors:** Louise Öhlund, Michael Ott, Malin Bergqvist, Sofia Oja, Robert Lundqvist, Mikael Sandlund, Ellinor Salander Renberg, Ursula Werneke

**Affiliations:** Research Registrar, Sunderby Research Unit – Psychiatry, Department of Clinical Sciences, Umeå University, Sweden; Consultant Physician, Department of Public Health and Clinical Medicine – Medicine, Umeå University, Sweden; Consultant Psychiatrist, Piteå Älvdals Hospital, Department of Psychiatry, Sweden; Consultant Psychiatrist, Department of Psychiatry, Sunderby Hospital, Sweden; Statistician, Research Unit, County Council of Norrbotten, Sweden; Professor of Psychiatry, Department of Clinical Sciences – Psychiatry, Umeå University, Sweden; Associate Professor of Psychiatry, Sunderby Research Unit – Psychiatry, Department of Clinical Sciences, Umeå University, Sweden

**Keywords:** Bipolar disorder, lithium, mood stabiliser, admission, hospitalisation

## Abstract

**Background:**

Currently, the evidence for lithium as a maintenance treatment for bipolar disorder type II (BD-II) remains limited. Guidelines commonly extrapolate recommendations for BD-II from available evidence for bipolar disorder type I (BD-I). Comparing the impact of lithium discontinuation is one way of assessing effectiveness in both groups.

**Aims:**

To compare the impact of lithium discontinuation on hospital admissions and self-harm in patients with BD-I or schizoaffective disorder (SZD) and patients with BD-II or other bipolar disorder.

**Method:**

Mirror-image study, examining hospital admissions within 2 years before and after lithium discontinuation in both patient groups. This study was part of a retrospective cohort study (LiSIE) into effects and side-effects of lithium for maintenance treatment of bipolar disorder as compared with other mood stabilisers.

**Results:**

For the whole sample, the mean number of admissions/patient/review period doubled from 0.44 to 0.95 (*P*<0.001) after lithium discontinuation. The mean number of bed days/patient/review period doubled from 11 to 22 (*P* = 0.025). This increase in admissions and bed days was exclusively attributable to patients with BD-I/SZD. Not having consulted with a doctor prior to lithium discontinuation or no treatment with an alternative mood stabiliser at the time of lithium discontinuation led to more admissions.

**Conclusions:**

The higher relapse risk in patients with BD-I/SZD suggests a higher threshold for discontinuing lithium than for patients with BD-II/other bipolar disorder. In patients with BD-II/other bipolar disorder, however, judged on the impact of discontinuation alone, lithium did not appear to prevent more severe depressive episodes requiring hospital admission.

Lithium remains the first choice as a maintenance treatment for bipolar disorder in the UK.^1^ Several large register studies have recently endorsed lithium as superior to other mood stabilisers, concerning the prevention of severe affective episodes, hospital admissions and suicide.^2–6^ Such register studies are useful for showing trends in healthcare use. However, without clinical, symptom-level data the distinction between different types of bipolar disorder can be difficult.^7^ This can limit the validity of findings.^5,7,8^ Current guidelines acknowledge that evidence for maintenance treatment is limited for bipolar disorder type II (BD-II). Therefore, many guidelines extrapolate their recommendation for BD-II from the available evidence for bipolar disorder type I (BD-I) and point out that treatment should be individualised.^1,9–11^ The impact of mixed features, when both manic or hypomanic and depressive symptoms occur at the same time or in close temporal succession, is also only poorly understood.^12^ In a previous study based on clinical data at symptom and treatment level, we explored the reasons why patients discontinued lithium treatment. We identified 561 episodes of lithium discontinuation with 922 individual reasons. In 62% of episodes the reason cited was adverse effects, in 44% psychiatric reasons and in 12% physical reasons interfering with lithium treatment.^13^ Here, using the same data source, we compare the impact of lithium discontinuation on clinical course and hospital admissions in patients with BD-I or schizoaffective disorder (SZD) and patients with BD-II or other bipolar disorder.

## Method

### Study design

This study was part of a retrospective (historical) cohort study (LiSIE) to explore effects and side-effects of lithium for the maintenance treatment of bipolar disorder as compared with other mood stabilisers. The LiSIE study was approved by the Regional Ethics Review Board at Umeå University, Sweden (DNR 2010-227-31M, DNR 2011-228-32M, DNR 2014-10-32M). The participants were informed about the nature of the study in writing and provided verbal informed consent. The consent was documented in our research files, dated and signed by the research worker who obtained the consent. In accordance with the ethics approval granted, for deceased patients, no consent was obtained.

### Participants

For LiSIE, we identified all patients in the Swedish region of Norrbotten, at least 18 years of age and who, according to ICD-10, had been diagnosed with bipolar disorder (F31) or SZD (F25) or who had been prescribed lithium as a mood stabiliser. Patients were eligible when they had received a diagnosis of bipolar disorder or SZD on at least two occasions, at least 6 months apart any time between 1997 and 2011. We retrospectively examined routine clinical data recorded until 31 December 2015 and performed the data extraction and validation in 2016 and 2017. The data was analysed in 2018.

Patients were potentially eligible for inclusion into this study when we found a lithium prescription discontinued according to the electronic prescription database. We included all patients, who (a) had discontinued lithium at any time between 1997 and 2013 for more than 7 days with the intention of stopping for good as indicated by the medical records, (b) had continuously received lithium for at least 2 years before stopping lithium, and (c) were available for follow-up for 2 years after stopping lithium. Some patients had discontinued lithium on several occasions. For the purpose of this study, we only considered the first episode of discontinuation preceded by 2 years of continuous lithium treatment. To achieve a perfect mirror, we excluded all patients whose review periods fell short of 2 years post-lithium discontinuation.

### Variable definitions and outcomes

The index event for our mirror-image study was lithium discontinuation. The pre- and post-mirror-image review periods, were both set to 2 years. We systematically abstracted the case records for each patient to obtain information regarding (a) baseline characteristics including diagnosis, (b) date of lithium discontinuation, and (c) length of lithium treatment before discontinuation. As outcomes, within 2 years before and after lithium discontinuation, we recorded hospital utilisation in terms of (a) number and type of hospital admissions, (b) type of affective episode at admission, and (c) bed days in hospital. When lithium was discontinued during an admission, we counted that admission in the pre-mirror period. We also compared the frequency of documented self-harm events before and after stopping lithium. All events of self-harm or suicide attempts, where a patient had received psychiatric, medical or surgical care in connection with the event, were recorded.

#### Diagnosis

In the medical records, clinicians had used diagnoses either according to DSM or ICD in their various editions. To establish the diagnoses used for our analysis, we considered diagnoses recorded in the medical records at four time points, (a) time of lithium start, (b) before lithium discontinuation, (c) at the end of the 2 years follow-up period after lithium discontinuation, and (d) last diagnosis before the end of our review period. From the four individual diagnoses, we then created a summary diagnosis as an approximation of what most likely the lifetime diagnosis would be according to DSM-5.^14^ We divided diagnoses into two categories, (a) BD-I/SZD and (b) BD-II/other bipolar disorder. The presence of a manic episode precluded patients from being allocated to the second group. In the category ‘other bipolar disorder’, we included patients with an explicitly recorded diagnosis of bipolar disorder, who (a) had not been given a diagnosis of BD-II and (b) had not experienced any manic episode (which would have lifted them into the BD-I category). This category included patients with a recorded diagnosis of BD not further specified and patients for whom the clinicians specified that a hypomanic episode had only appeared after taking antidepressant medication. We also checked whether patients ever had mixed features in a mood-disorder episode (mixed features) or rapid cycling. The final diagnoses were validated by at least two researchers (LÖ, MB, SO, UW).

#### Lithium exposure

Patients with lithium prescriptions in the LiSIE cohort had to have at least two lithium serum concentrations >0.2 mmol/L recorded in the laboratory database to count as truly exposed. For this particular study, patients needed to have been treated with lithium for at least 2 years before stopping lithium. There was no minimum requirement for a lithium serum concentration. Instead, we adjusted for mean lithium serum concentration in the 2-year period before lithium discontinuation in our multiple linear regression analysis. We also confirmed the starting point of lithium treatment as documented in the medical records, going back as far as 1967.

#### Lithium discontinuation

We included all episodes for which the medical records indicated that lithium was discontinued with the intention to stop for good. Since lithium discontinuation could precede cancellation of the prescription, we confirmed from the medical records the point of time of lithium discontinuation as accurately as possible. We distinguished between rapid discontinuation, within 0 and 14 days, and gradual discontinuation, where lithium was tapered over more than 14 days. We also checked the records for the reasons for lithium discontinuation and if patients had consulted a doctor before.

#### Mood stabilising treatment at the point of lithium discontinuation and during the post-mirror period

We also compared mood stabiliser treatment after lithium discontinuation as a potential major confounder for our outcomes. We checked the medical notes for use of alternative mood stabilisers – olanzapine, quetiapine, aripiprazole, risperidone, valproate, lamotrigine, carbamazepine and topiramate – at the point of lithium discontinuation, at 3 months and 2 years after lithium discontinuation. We defined alternative mood stabiliser treatment as stable by proxy of having been treated with the same mood stabiliser at 3 months and 2 years after lithium discontinuation. We also checked for lithium reinstatement.

#### Alcohol and/or substance use disorder

We screened the medical notes for alcohol and/or substance use disorder within 2 years before and after lithium discontinuation. Alcohol and/or substance use disorder was recorded when (a) there was an explicit diagnosis according to DSM or ICD in their various editions, or (b) when there was an explicit reference in the medical notes.

### Control for bias

Of all patients approached, 75% consented to inclusion in the LiSIE study. In accordance with the ethical approval granted, we controlled for selection bias, comparing age, gender, maximum recorded serum lithium and creatinine levels as key parameters, available in anonymised form. We did not find any significant difference between participating and non-participating patients. To minimise observer and recording bias, all data was abstracted by two separate reviewers.

### Statistical analysis

We conducted a descriptive analysis, establishing the frequency of all variables in our database. Means and standard deviations were calculated for continuous data. Frequencies and percentages were calculated for categorical data. We then stratified further according to type of bipolar disorder. For group comparisons of continuous variables, we used paired *t*-test when group sizes were sufficiently large (≥40 in either group) as this test is fairly robust against violations of the normal distribution assumption.^15^ For smaller group sizes, we used Wilcoxon signed rank test. For categorical variables, we used McNemar's and Pearson's χ^2^-tests.

We conducted multiple linear regression analyses in order to adjust for possible confounders in the analysis of the association between the change in hospital admissions and bed days and diagnostic group, BD-I/SZD versus BD-II/other bipolar disorder. We created three models with the following outcomes (a) change in total number of admissions, (b) change in number of compulsory admissions only, and (c) change in number of bed days. We tested for factors identified in the published literature:^9,13,16,17^ age, mixed status, lithium concentration, mood stabiliser treatment at lithium discontinuation, post-mirror stable mood stabiliser treatment, rapid or gradual withdrawal, consulted with a doctor before discontinuing, alcohol and/or substance use disorder. Effects were reported as estimated marginal means, which give the mean increase in unit of outcome, number of admissions or bed days, according to factors under study.

Kaplan–Meier plots were used to map the time periods from stopping lithium to first admission after lithium discontinuation. To account for type of bipolar disorder and speed of lithium discontinuation, we created four separate survival functions, comparing (a) BD-I/SZD versus BD-II/other bipolar disorder, (b) rapid versus gradual lithium discontinuation for the whole group, (c) rapid versus gradual lithium discontinuation for patients with BD-I/SZD only and (d) rapid versus gradual lithium discontinuation for patients with BD-II/other bipolar disorder only. We tested potential differences between groups using the log rank test. Throughout, the statistical significance level was set to *P*<0.05. The statistical analysis was conducted with IBM SPSS Statistics version 25.

## Results

### Participant characteristics

**Fig. 1:**
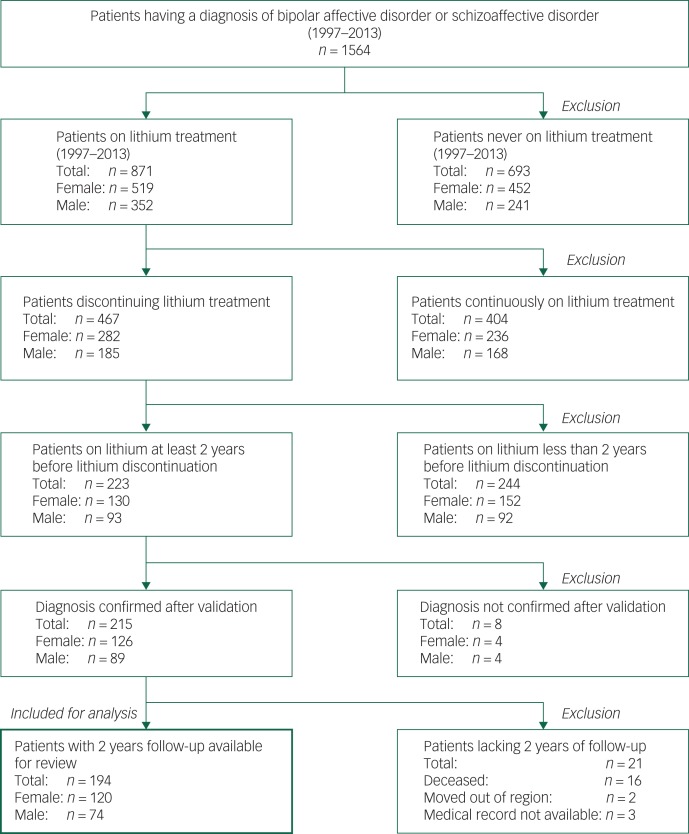
Identification of study sample.

Of 1564 eligible patients, 871 (56%) had been treated with lithium during the study period. There were 467 (54%) patients who discontinued lithium on at least one occasion with the intention of stopping for good. In total, 194 patients met the inclusion criteria (Fig. 1); their baseline characteristics are described in Table 1. The mean lithium concentration over the 2 years before lithium discontinuation was 0.61 (s.d. = 0.19) mmol/L, based on 1734 individual measurements in the whole sample (Table 1).

**Table 1:** Baseline characteristics

	Total sample	BD-I/SZD	BD-II/other bipolar disorder^a^	*P*
All, *n* (%)	194	100 (51.5)	94 (48.5)	
Gender, *n* (%)				
Women	120 (61.9)	55 (55.0)	65 (69.1)	0.043
Men	74 (38.1)	45 (45.0)	29 (30.9)	
Age at lithium discontinuation				
Mean (s.d.)	52.5 (14.4)	54.9 (14.0)	50.1 (14.5)	0.019
<65 years, *n* (%)	153 (78.9)	76 (76.0)	77 (81.9)	0.313
≥65 years, *n* (%)	41 (21.1)	24 (24.0)	17 (18.1)	
Time on lithium before discontinuation (years)				
Mean (s.d.)	9.4 (8.0)	11.5 (8.6)	7.2 (6.6)	<0.001
≤5 years, *n* (%)	87 (44.8)	32 (32.0)	55 (58.5)	<0.001
>5 years, *n* (%)	107 (55.2)	68 (68.0)	39 (41.5)	
Mixed features in a mood-disorder episode ever,^b^ yes *n* (%)	56 (28.9)	35 (35.0)	21 (22.3)	0.052
Lithium concentration (mmol/L) during the pre-mirror period				
Mean (s.d.)	0.61 (0.2)	0.61 (0.2)	0.61 (0.2)	0.845
≥1.5, *n* (%)	1 (0.5)	0 (0.0)	1 (1.1)	
>0.8 to <1.5, *n* (%)	22 (11.3)	10 (10.0)	12 (12.8)	
0.6–0.8, *n* (%)	82 (42.3)	46 (46.0)	36 (38.3)	
≥0.4 to <0.6, *n* (%)	65 (33.5)	33 (33.0)	32 (34.0)	
<0.4, *n* (%)	23 (11.9)	11 (11.0)	12 (12.8)	
Not available, *n* (%)	1 (0.05)	0 (0.0)	1 (1.1)	
Other mood stabiliser at the time of lithium discontinuation^c^, yes *n* (%)	72 (37.1)	36 (36.0)	36 (38.3)	0.741
Stable treatment with an alternative mood stabiliser during post-mirror period,^d^ yes *n* (%)	65 (33.5)	35 (35.0)	30 (31.9)	0.649
Lithium reinstated during post-mirror period, yes *n* (%)	61 (31.4)	41 (41.0)	20 (21.3)	0.003
Speed of lithium withdrawal, *n* (%)				
Gradual, >14 days	54 (27.8)	22 (22.0)	32 (34.0)	0.048
Rapid, ≤14 days	136 (70.1)	77 (77.0)	59 (62.8)	
Unknown	4 (2.1)	1 (1.0)	3 (3.2)	
Reason for lithium discontinuation *n* (%)				
Adverse effects/physical health reasons only	109 (56.2)	59 (59.0)	50 (53.2)	0.366
Psychiatric reasons only	52 (26.8)	22 (22.0)	30 (31.9)	
Adverse effects/physical health reasons and psychiatric reasons combined	27 (13.9)	13 (13.0)	14 (14.9)	
Unknown	6 (3.1)	6 (6.0)	0 (0.0)	
Patient consulted doctor before lithium discontinuation, yes *n* (%)	123 (63.4)	60 (60.0)	63 (67.0)	0.310
Substance/alcohol use disorder during post-mirror period, yes *n* (%)	16 (8.2)	4 (4.0)	12 (12.8)	0.027

All proportions are rounded to one decimal place. Percentages may not always add up perfectly to 100%.

BD-I, bipolar disorder type I; BD-II, bipolar disorder type II; SZD, schizoaffective disorder.

a. Unspecified bipolar disorder or subgroup specified otherwise.

b. Occurrence of an episode with mixed features or rapid cycling ever.

c. Valproate, lamotrigine, carbamazepine, topiramate, aripiprazole, quetiapine, olanzapine, risperidone.

d. Treatment with the same kind of mood stabiliser at 3 months and at 24 months after lithium discontinuation, for example valproate, lamotrigine, carbamazepine, topiramate, aripiprazole, quetiapine, olanzapine, risperidone.

### Treatment with mood stabilisers at the point of lithium discontinuation and during the post-mirror period

At the point of lithium discontinuation, 36% of patients with BD-I/SZD and 38% of patients with BD-II/other bipolar disorder received alternative mood stabiliser treatment (*P* = 0.741) (Table 1). Three months after lithium discontinuation, 50% of patients with BD-I/SZD and 38% of patients with BD-II/other bipolar disorder were treated with an alternative mood stabiliser (*P* = 0.101). Two years after lithium discontinuation, 51% of patients with BD-I/SZD and 46% of patients with BD-II/other bipolar disorder, were treated with an alternative mood stabiliser (*P* = 0.464). During the post-mirror period, 35% of patients with BD-I/SZD and 32% of patients with BD-II/other bipolar disorder were considered to have a stable treatment with an alternative mood stabiliser (*P* = 0.649). In total, 31% of patients reinstated lithium at some point during the post-mirror period. Patients with BD-I/SZD restarted their lithium treatment significantly more often than patients with BD-II/other bipolar disorder (*P* = 0.003) (Table 1).

### Change in number of admissions within 2 years before and after lithium discontinuation

In the whole sample, 37 patients had 86 admissions before, and 79 patients had 185 admissions after lithium discontinuation. The number of admissions increased for about 33% of patients and decreased for 10%. For 57%, there was no change. The mean number of admissions/patient/review period increased significantly (*P*<0.001). The number of compulsory admissions increased for 13% of patients and decreased for 2% after lithium discontinuation. For 85%, there was no change. The mean number of compulsory admissions/patient/review period also increased significantly (*P*<0.001). There were significantly more admissions for all types of episodes after lithium discontinuation, including mood episodes with mixed features (Table 2).

**Table 2:** Hospital admissions and bed days within 2 years before and after lithium discontinuation

	Total sample (*n* = 194)			BD-I/SZD (*n* = 100)			BD-II/other bipolar disorder^a^ (*n* = 94)		
	Before	After	*P*	Before	After	*P*	Before	After	*P*
Admissions									
Patients with admissions, *n* (%)	37 (19.1)	79 (40.7)	<0.001	18 (18.0)	56 (56.0)	<0.001	19 (20.2)	23 (24.5)	0.454
Total admissions, *n*	86	185		33	130		53	55	
Admissions/patient/review period,^b^ mean (s.d.)	0.44 (1.28)	0.95 (1.58)	<0.001	0.33 (0.94)	1.30 (1.64)	<0.001	0.56 (1.55)	0.59 (1.43)	0.906
Voluntary admissions, *n*	80	144		30	90		50	54	
Voluntary admissions/patient/review period, mean (s.d.)	0.41 (1.25)	0.74 (1.38)	0.003	0.30 (0.94)	0.90 (1.37)	<0.001	0.53 (1.52)	0.57 (1.38)	0.808
Compulsory admissions, *n*	6	41		3	40		3	1	
Compulsory admissions/patient/review period, mean (s.d.)	0.03 (0.17)	0.21 (0.64)	<0.001	0.03 (0.17)	0.40 (0.84)	<0.001	0.03 (0.18)	0.01 (0.10)	0.320
Type of affective episode recorded at admission^c^									
Mania/hypomania, *n*^d^	11	64		10	64		1	0	
Mania/hypomania/patient/review period, mean (s.d.)	0.06 (0.31)	0.33 (0.79)	<0.001	0.10 (0.41)	0.64 (1.0)	<0.001	0.01 (0.10)	0.00 (0.00)	0.320
Mania only, *n*	7	51		7	51		0	0	
Mania/patient/review period, mean (s.d.)	0.04 (0.21)	0.26 (0.69)	<0.001	0.07 (0.29)	0.51 (0.89)	<0.001	0.00 (0.00)	0.00 (0.00)	−
Hypomania only, *n*	4	13		3	13		1	0	
Hypomania/patient/review period, mean (s.d.)	0.02 (0.23)	0.07 (0.37)	0.139	0.03 (0.30)	0.13 (0.51)	0.096	0.01 (0.10)	0.00 (0.00)	0.320
Depression, *n*	38	71		9	35		29	36	
Depression/patient/review period, mean (s.d.)	0.20 (0.73)	0.37 (1.18)	0.035	0.09 (0.43)	0.35 (1.06)	0.009	0.31 (0.94)	0.38 (1.30)	0.561
Mood-disorder episode with mixed features, *n*	2	11		2	9		0	2	
Mood-disorder episode with mixed features/patient/review period, mean (s.d.)	0.01 (0.10)	0.06 (0.27)	0.020	0.02 (0.14)	0.09 (0.35)	0.052	0.00 (0.00)	0.02 (0.15)	0.158
Other reason, *n*^e^	35	39		12	22		23	17	
Other reason/patient/review period, mean (s.d.)	0.18 (0.87)	0.20 (0.67)	0.776	0.12 (0.54)	0.22 (0.69)	0.253	0.24 (1.11)	0.18 (0.66)	0.587
Bed days									
Bed days, *n*	2218	4240		1018	3196		1200	1044	
Bed days/patient/review period, mean (s.d.)	11.4 (39.1)	21.9 (60.7)	0.025	10.2 (29.1)	32.0 (72.0)	0.005	12.8 (47.7)	11.1 (43.6)	0.733

BD-I, bipolar disorder type I; SZD, schizoaffective disorder; BD-II, bipolar disorder type II.

a. Unspecified bipolar disorder or subgroup specified otherwise.

b. Period 2 years before and after lithium discontinuation.

c. Only main reason for admission given.

d. In BD-II/other bipolar disorder only hypomania.

e. Paranoid psychosis, unspecified psychosis, anxiety, insomnia, drug adjustments, substance or alcohol misuse.

### Change in number of admissions according to type of disorder

For patients with BD-I/SZD, the number of admissions increased for 50% of patients and decreased for 7%. For 43%, there was no change. The number of admissions increased from 33 to 130. The mean number of admissions/patient/review period increased significantly (*P*<0.001) (Table 2). Further analysis showed that this increase was irrespective of whether patients ever had experienced mixed features or not (*P*<0.001 for both). There were significantly more admissions for both manic (*P*<0.001) and depressive episodes (*P* = 0.009). For patients with BD-I/SZD, the number of admissions increased significantly, irrespective of whether lithium was reinstated (*P*<0.001) or not (*P* = 0.009).

For patients with BD-II/other bipolar disorder, the number of admissions increased for 16% of patients and decreased for 13%. For 71%, there was no change. The number of admissions increased from 53 to 55. The mean number of admissions/patient/review period did not change significantly after lithium discontinuation, regardless of reason for admission (Table 2). Neither did this number change for patients with mixed or without mixed features ever (*P* = 0.863 and *P* = 0.545, respectively). For patients with BD-II/other bipolar disorder, there was no significant change in number of admissions, irrespective of whether lithium was reinstated (*P* = 0.667) or not (*P* = 0.669).

For patients with BD-I/SZD, the multiple linear regression model showed that the mean number of admissions after lithium discontinuation was significantly higher compared with BD-II/other bipolar disorder (*P*<0.001). Not having consulted with a doctor prior to lithium discontinuation (*P* = 0.032) was another factor associated with significantly more admissions after lithium discontinuation. However, being treated with an alternative mood stabiliser at the time of lithium discontinuation was associated with significantly fewer admissions (*P* = 0.012) (Table 3). The mean number of compulsory admissions in patients with BD-I/SZD, compared with BD-II/other bipolar disorder, was also higher (0.38, 95% CI 0.18–0.57, *P*<0.001). Having consulted with a doctor prior to lithium discontinuation was associated with fewer compulsory admissions after lithium discontinuation (−0.29, 95% CI −0.50 to −0.08, *P* = 0.007). In this model, being treated with an alternative mood stabiliser at the time of lithium discontinuation was not statistically significant (−0.12, 95% CI −0.36 to 0.11, *P* = 0.308) (data not shown in detail).

**Table 3:** Factors associated with changes in the number of admissions and bed days after lithium discontinuation

Parameter	Admissions				Bed days			
	Mean difference^a^	95% CI		*P*	Mean difference^a^	95% CI		*P*
		Lower	Upper			Lower	Upper	
Gender								
Men	−0.122	−0.637	0.392	0.639	−3.234	−22.675	16.208	0.743
Women (baseline)								
Type of disorder								
BD-I/SZD	1.011	0.502	1.520	<0.001	23.946	4.707	43.184	0.015
BD-II/other bipolar disorder (baseline)								
Age at lithium discontinuation, mean	−0.003	−0.022	0.015	0.717	−0.106	−0.798	0.585	0.762
Mixed episode ever								
Yes	0.123	−0.426	0.673	0.658	18.717	−2.052	39.485	0.077
No (baseline)								
Lithium concentration (mmol/L) within 2 years before lithium discontinuation	0.892	−0.432	2.216	0.185	1.794	−48.281	51.870	0.944
Other mood stabiliser at the time of lithium discontinuation								
Yes	−0.804	−1.433	−0.176	0.012	−18.178	−41.953	5.597	0.133
No (baseline)								
Stable treatment with an alternative mood stabiliser during post-mirror period								
Yes	0.321	−0.330	0.972	0.332	−4.056	−28.668	20.556	0.745
No (baseline)								
Speed of withdrawal								
Gradual, >14 days	0.267	−0.291	0.824	0.346	13.436	−7.635	34.506	0.210
Rapid, ≤14 days (baseline)								
Patient consulted doctor before lithium discontinuation								
Yes	−0.615	−1.175	−0.054	0.032	−19.780	−40.963	1.403	0.067
No (baseline)								
Substance/alcohol use disorder within 2 years after lithium discontinuation								
Yes	0.696	−0.197	1.589	0.126	14.811	−18.951	48.573	0.388
No (baseline)								

BD-I, bipolar disorder type I; SZD, schizoaffective disorder; BD-II, bipolar disorder type II; other bipolar disorder, unspecified bipolar disorder or subgroup specified otherwise.

a. Of estimated marginal means.

### Change in number of bed days within 2 years before and after lithium discontinuation

In the whole sample, the number of bed days increased for 33% of patients and decreased for 13%. For 54%, there was no change. The total number of bed days increased from 2218 to 4240 after lithium discontinuation. Mean number of bed days/patient/review period increased significantly (*P* = 0.025) (Table 2).

### Change in number of bed days according to type of disorder

For patients with BD-I/SZD, the number of bed days increased for 49% of patients and decreased for 12%. For 39%, there was no change. The number of bed days increased from 1018 to 3196. Mean number of bed days/patient/review period increased significantly (*P* = 0.005) (Table 2). Further analysis showed that this increase was irrespective of whether patients ever had experienced mixed features or not (*P* = 0.001 and *P* = 0.024). For patients with BD-I/SZD who reinstated lithium, the number of bed days increased significantly (*P*<0.001). For those who did not reinstate lithium, there was no change (*P* = 0.209).

For patients with BD-II/other bipolar disorder, the number of bed days increased for 16% of patients and decreased for 14%. For 70%, there was no change. The number of bed days decreased from 1200 to 1044. For patients with BD-II/other bipolar disorder, mean number of bed days did not change significantly after lithium discontinuation (*P* = 0.733) (Table 2). Neither did this number change for patients with or without mixed features ever (*P* = 0.575 and *P* = 0.444, respectively). For patients with BD-II/other bipolar disorder, there was no significant change in number of bed days, irrespective of whether lithium was reinstated (*P* = 0.767) or not (*P* = 0.920).

The multiple linear regression model showed that after lithium discontinuation, compared with BD-II/other bipolar disorder, the mean number of bed days was significantly higher for patients with BD-I/SZD (*P* = 0.015). In this model, not having consulted with a doctor prior to lithium discontinuation (*P* = 0.067) and being treated with an alternative mood stabiliser at the time of lithium discontinuation was not statistically significant (*P* = 0.133) (Table 3).

### Rapid or gradual discontinuation of lithium

In the whole sample, 70% of patients were ‘rapid discontinuers’. Gradual lithium withdrawal was significantly more common among patients with BD-II/other bipolar disorder compared with patients with BD-I/SZD (*P* = 0.048) (Table 1). In the group of rapid discontinuers, significantly fewer patients consulted with a doctor before stopping lithium (*P*<0.001). At the time of lithium discontinuation, treatment with other mood stabilisers did not differ significantly between rapid and gradual discontinuers (*P* = 0.071). The adjusted regression model showed no significant difference in number of hospital admissions or bed days in relation to the speed of lithium discontinuation (Table 3).

**Fig. 2:**
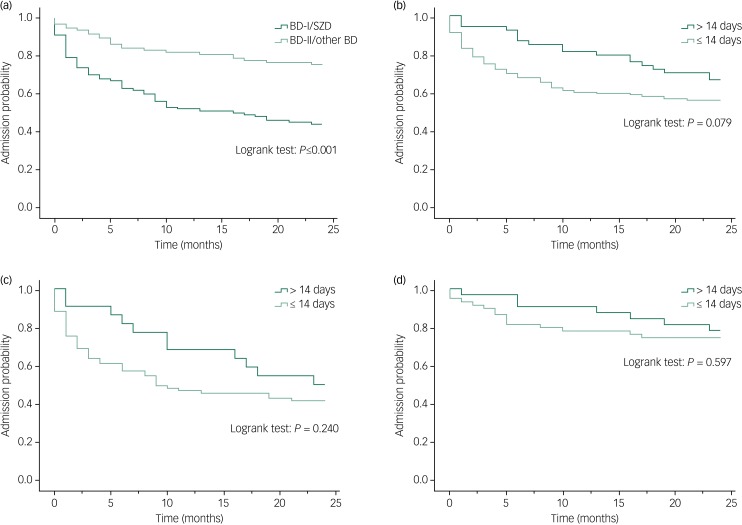
‘Survival’ time to first psychiatric hospital admission within 2 years after lithium discontinuation.

The Kaplan–Meier plots, which mapped the time periods from lithium discontinuation to first admission for different subgroups, are shown in Fig. 2. The mean ‘survival’ time to first admission after lithium discontinuation was 14 months (95% CI 11.7–15.7) for patients with BD-I/SZD and 20 months (95% CI 18.3–21.5) for patients with BD-II/other. There was a significant difference between both survival curves (*P*<0.001), indicating a worse course for patients with BD-I/SZD (Fig. 2(a)). The survival curves did not show any significant difference between rapid or gradual discontinuers, neither for the whole sample (*P* = 0.079) (Fig. 2b), nor for BD-I/SZD (*P* = 0.240) (Fig. 2(c)), nor for patients with BD-II/other bipolar disorder (*P* = 0.597) (Fig. 2(d)).

( a) Probability of admission at time *t* or later, stratified by type of subgroup; (b) probability of admission at time *t* or later, stratified by speed of withdrawal; (c) probability of admission at time *t* or later, stratified by bipolar disorder (BD)-I/ schizoaffective disorder (SZD) and speed of withdrawal; (d) probability of admission at time *t* or later, stratified by BD-II/other bipolar disorder and speed of withdrawal.

### Self-harm after lithium discontinuation

In the pre-mirror period, six patients had eight episodes of self-harm. In the post-mirror period, 12 patients had 18 episodes of self-harm. This did not constitute a significant change (*P* = 0.180). Neither were there significantly more episodes of self-harm per patient (*P* = 0.069). However, men (*P* = 0.046) but not women (*P* = 0.249) had significantly more episodes of self-harm after lithium discontinuation. Regarding diagnostic subgroups, only patients with BD-I/SZD who had ever experienced mixed features had significantly more episodes of self-harm (*P* = 0.034) after lithium discontinuation.

## Discussion

### Main findings

To our knowledge, this is the first study examining the effects of lithium discontinuation for different subtypes of bipolar disorder with a mirror-image design. The main finding of our study is that lithium discontinuation led to a significant increase in admissions overall, compulsory admissions and bed days in the post-mirror period. Based on the costs of a bed day in the three hospitals included in our study in 2018, this would have corresponded to additional hospital costs of 12 million Swedish crowns (1.4 million US dollars or 1.1 million British pounds, currency conversion rates as of 31 December 2018).

The increase in hospital utilisation was solely attributable to patients with BD-I/SZD. In this group, both patients with or without mixed features experienced a similar increase in admissions and bed days. Yet, patients with mixed features had more episodes of self-harm. After lithium discontinuation, patients with BD-I/SZD also had a significant shorter time to admission than patients with BD-II and other bipolar disorder. A comparable proportion of patients with BD-I/SZD and BD-II/other bipolar disorder used mood stabilisers after lithium discontinuation. Therefore, the increase in hospital utilisation could not be explained by less use of mood stabilisers in patients with BD-I/SZD after lithium discontinuation.

Our findings suggest that in patients with BD-I/SZD, lithium discontinuation comes at a considerable cost, not only in terms of deterioration of illness but also resources. In patients with BD-II/other bipolar disorder, however, judged on the impact of discontinuation alone, lithium did not appear to prevent more severe depressive episodes requiring hospital admissions. If we considered BD-I/SZD a more serious illness than BD-II/other bipolar disorder based on the criterion of mania, which patients with BD-II by definition cannot experience,^18^ our findings might not be considered surprising. But as patients acted as their own controls, illness severity should not affect the results. We found a huge effect size for BD-I/SZD but no significant effect for BD-II/other bipolar disorder. In any event, in terms of suicidal attempts, BD-II seems to be an equally severe condition as BD-I.^18,19^ BD-II may even be more severe in terms of chronicity, impairment from depressive symptoms and subthreshold symptoms.^18^ This was also observed in our study.

### Comparison with other studies

#### Proportion of patients discontinuing lithium

In our study, 54% of patients discontinued lithium during our observation period. This is in line with other studies; medication non-adherence rates of 50% have been reported for patients with bipolar disorder.^20^ For lithium, reported non-adherence rates have varied widely, from 6^21^ to 58%.^22^ Adherence estimates depend on a variety of factors, including length of the observation period, patient population sampled and prospective or retrospective study design. The means by which non-adherence is measured are also important: prescription counts, medicine possession ratios, patient self-reports, physician estimates or lithium concentrations may all yield different results.^23,24^

#### Rapid or gradual lithium discontinuation

Previous studies have shown that gradual instead of rapid discontinuation can reduce manic and depressive relapses and even suicidal risk. Interestingly, the advantages of gradual discontinuation were greater in BD-II than BD-I, both in terms of longer time until first relapse and longer periods of stability.^16,17^ After lithium discontinuation, relapse into mania tends to occur sooner than relapse into depression and can be delayed by gradual discontinuation.^16,17,25^ In our study, speed of lithium discontinuation was not significantly associated with change in the number of admissions or bed days. However, not having consulted with a doctor was associated with an increase in numbers of admissions. Being treated with an alternative mood stabiliser at time of lithium discontinuation also protected against readmission. This highlights the importance of continued regular contact with patients using lithium maintenance therapy and continued engagement after lithium discontinuation.^13^ However, as pointed out above, the rate of lithium discontinuation might not have affected the number of future admissions, but rather the time to first admission after lithium discontinuation.^16^ In our study, survival curves did not show any significant difference between rapid or gradual discontinuers, neither for the whole group, nor for the BD-I/SZD and BD-II/other bipolar disorder subgroups.

#### Lithium as a maintenance treatment for BD-II

Several large cohort and register studies have shown the superiority of lithium for the treatment of bipolar disorder. However, most have not distinguished between the different types.^2,4,6,26,27^ Subtypes of bipolar disorder may not be part of the register data informing large cohort studies.^4^ Misclassification can become a problem if different diagnostic systems or different versions of the same diagnostic system have been used. In 2018, based on a systematic review of naturalistic observational studies, Kessing *et al* suggested the superiority of lithium in real-life settings.^28^ Of 11 studies included, only two stratified their results according to BD-I or BD-II.^29,30^ One of these suggested that patients with BD-II were more likely to be lithium responders than patients with BD-I.^29^ The other one concluded that lithium had some advantage compared with other stabilising treatment in both BD-I and BD-II.^30^ A further study, not included in that systematic review, found that ‘affective morbidity, as reflected in the portion of time ill during lithium treatment, was significantly lower in BD-II than BD-I’.^31^ Our own study of reasons for discontinuation of lithium treatment in 468 patients showed that patients with BD-II/other bipolar disorder more often stopped lithium as a result of a perceived lack of effect. Time on lithium was also significantly shorter.^13^

Soares-Weiser *et al* conducted an extensive systematic review and meta-analysis of randomised controlled or quasi-randomised controlled trials on the effectiveness of various mood stabilisers. They suggested that lithium might be less effective for BD-II, but ultimately the evidence was too weak to arrive at a firm conclusion.^32^ Another recently updated meta-analysis of randomised controlled studies explored relapse prevention in patients with bipolar disorder, comparing lithium against placebo and anticonvulsants.^33,34^ Lithium was more effective than placebo for the prevention of overall mood and manic episodes. Lithium was also more effective than anticonvulsants for the prevention of manic, but not of overall mood or depressive episodes. Fourteen studies were included. However, only two trials explicitly concerned patients with BD-II.^35,36^ Two further trials included a fraction of patients with BD-II^37^ and bipolar disorder not otherwise specified.^38^ Therefore, the results of this meta-analysis are mainly applicable to BD-I. Two more recent trials compared lithium with antidepressants in patients with BD-II. In both trials, there was no difference in relapse rate.^39,40^ Yet, concerns remain that antidepressants, unopposed by mood stabilisers, increase the risk of manic episodes.^41,42^ In view of the sparse evidence, treatment recommendations for BD-II mainly reflect findings from BD-I studies. Most guidelines also stress the importance of taking the predominant polarity into account.^9–11,43^ Only the Canadian guideline distinguishes clearly between BD-I and BD-II, recommending quetiapine, lithium or lamotrigine as first-line agents for the maintenance treatment of BD-II disorder.^42^

#### Lithium as a treatment for bipolar disorder with mixed features

At present, the impact of mixed features on treatment outcomes of bipolar disorder is only poorly understood.^9^ Most maintenance treatment trials concern mixed features in the context of BD-I but not BD-II.^44^ Guidelines tend to agree that antidepressants should not be used in mixed states.^1,9,10,41,42^ Evidence concerning mood stabilisers as maintenance therapies for patients with mixed features is still virtually absent.^12^

#### Lithium for the prevention of suicide and self-harm in patients with BD-II and mixed states

It has been suggested that the risk of suicide in BD-II is higher than in BD-I. The reasons for this are not quite clear. A higher occurrence of agitated depression and anxiety may play a role.^45^ A Finnish cohort study monitored the incidence of suicide attempts in 177 patients with bipolar disorder for 5 years. In this study, type of bipolar disorder did not emerge as a predictor of suicide attempts. The highest incidence of suicide attempts was seen during mixed states.^46^ In another study of 3099 out-patients with major depressive disorder or bipolar disorder, mixed features also increased the risk of suicidal acts.^47^ Yet, a third study of 290 patients with bipolar disorder found that depressive symptoms carried an increased risk of suicidal ideation but mixed symptoms did not contribute additionally.^48^

Few studies have explicitly explored the role of lithium in the prevention of suicide and self-harm in patients with BD-II and mixed states. A recent meta-analysis found a substantial risk reduction of suicide and suicide attempts among patients with bipolar disorder overall during long-term lithium treatment.^49^ As suicide and suicide attempts are relatively rare events, it is difficult to power trials sufficiently to examine these outcomes. This makes it even more difficult to stratify by type of bipolar disorder. In an 8-year prospective Swedish register study of 50 000 patients, lithium significantly reduced suicide-related events for patients with BD-II, but not with BD-I or mixed bipolar disorder.^5^ In our study, patients with BD-I /SZD with mixed features had significantly more episodes of self-harm after lithium discontinuation.

### Strengths

The mirror-image design allowed us to conduct a quasi-experimental observational study with lithium discontinuation as the intervention. The pre/post design allowed individuals acting as their own controls, thereby minimising confounding at an individual level.^50,51^ Our study was not based on register data but real-life detailed clinical data at symptom and treatment level going back as far as 1967. In most randomised controlled trials, retention in a 2-year study may not exceed 10%.^9^ In our study, of all the patients who received lithium for at least 2 years prior to discontinuation, 90% were also available for follow-up for 2 years after discontinuation. A further strength was the high rate of consent for participation into the study. Age and gender distribution of consenting and non-consenting patients was similar. Therefore, the likelihood of selection bias is low. We could validate the diagnoses from the case records. This allowed distinction between the various types of bipolar disorder, adjustment for mixed features and exclusion of other diagnoses including schizophrenia. We established lithium treatment status, not only by prescription but also by lithium serum concentrations. Access to the medical records allowed us to determine the time point of lithium discontinuation much more accurately. Our main outcomes, number of hospital admissions and bed days, were easily verifiable in the medical records. For the detection of self-harm episodes, integrated records from the other specialist services within the region were available for review.

### Limitations

Our study had a retrospective observational design using patients as their own controls. We did not compare patients who discontinued lithium with a ‘true’ control group of patients who remained on lithium. The non-randomised design limited our ability to make direct comparisons between lithium and other mood stabilisers. As in other studies,^52,53^ women had higher rates of BD-II in our study. But this is unlikely to have influenced our findings. Within the two diagnostic groups, there were no gender differences concerning hospital utilisation. Besides, men and women tend to respond similarly to lithium.^54^ As we only included patients who had taken lithium for at least 2 years before stopping, it is possible that the study was enriched by lithium responders. However, from our previous study of reasons for lithium discontinuation based on the same sample, we know that patients stayed on average 3.6 (s.d. = 6.1) years on lithium before stopping.^13^ Hence enrichment is unlikely to have played a major role.

For this particular study, we did not require any minimum serum concentration for the 2 years before lithium discontinuation. The mean lithium concentration turned out to be ≥0.6 mmol/L in 54% and ≥0.4 mmol/L in 88% of patients. Thus, the results would have been unlikely to differ if we had employed a specific lithium cut-off point. In our multiple linear regression analysis, lithium concentration was not significantly associated with a change in number of admissions or bed days.

The quality of our data depended on the quality of the information in the medical notes. This also held true for the recording of diagnosis and type of mixed features. But as we had access to data at symptom level, we judge the potential for misclassification to be less than in other observational studies based on register data. The mirror-image design minimised confounding at an individual level. However, there might have been other changes during the post-mirror period. For example, patients who discontinued lithium could also have stopped all other medications. Therefore, we checked treatment with mood stabilisers at the point of lithium discontinuation and in the post-mirror period.

The retrospective observational study design did not allow us to detect changes in mental status or functioning in daily life that did not lead to admission. Admission to hospital as the primary outcome criteria means that mild to moderately severe episodes will most likely be missed completely. This holds especially true for individuals with BD-II; hypomanic episodes rarely result in hospital admission. In some countries such as the USA, an admission to hospital may by definition change the diagnosis from hypomania to mania. Thus, for patients with BD-II, the pre–post lithium discontinuation design only makes it possible to compare hospital admission rates for depressions. But depressive episodes tend to affect quality of life much more than hypomanic episodes.^55^ Therefore, we consider the criterion of admissions/readmissions a valid outcome parameter, which has also been used in many other studies.^4,6,25^

As we did not find an effect of lithium discontinuation for patients with BD-II/other bipolar disorder, the question arises whether our study was underpowered. However, there was a significant change in the number of admissions and bed days for patients with BD-I/SZD. As the sample sizes for both BD-I/SZD and BD-II/other bipolar disorder were similar we would not expect the sample sizes to be insufficient in either case. Yet, the sample sizes in our study were insufficient to assess suicide as an outcome. Neither were the sample sizes sufficient to explore further factors associated with self-harm after lithium discontinuation. For the same reason, a more detailed examination of polarity of first admission after lithium discontinuation and subsequent lithium reinstatement were factors beyond the scope of this mirror-image study. We intend to take this up in future work based on the LiSIE study.

### Implications for practice

Lithium discontinuation in patients with BD-I/SZD comes at a cost of deteriorated mental health and increased hospital utilisation. In this group, risk of self-harm may particularly increase in men and patients with mixed features. In patients with BD-II/other bipolar disorder, judged on the impact of discontinuation alone, lithium did not appear to prevent more severe depressive episodes requiring hospital admissions. The role of lithium in the treatment of BD-II and prevention of suicide and self-harm irrespective of BD type requires further investigation. The higher relapse risk in patients with BD-I/SZD points towards a need to apply a higher threshold for lithium discontinuation for this group than for patients with BD-II/other bipolar disorder. Ultimately though, the decision to stop lithium has to be individualised for each patient. Our findings identify a need for more clinical research to enable treatment guidelines to distinguish the various types of bipolar disorder.

## Data Availability

The data-sets generated and/or analysed during the current study are not publicly available due to lack of ethics committee permission and not having been part of the consent process. The structure of the data-set and the coding specification are available from the authors. Any other reasonable request will be raised with the regional ethics committee and healthcare provider.
